# An anoikis-based risk model predicts outcomes and is associated with the immune microenvironment in adrenocortical carcinoma

**DOI:** 10.3389/fmolb.2026.1779180

**Published:** 2026-06-11

**Authors:** Juan Cao, Ming Xu, Wenjun Zhou, Shuxi Zhong, Xiaoxuan Cao, Huiping Xie, Zhiming Shen

**Affiliations:** 1 Department of Laboratory Medicine, The First Affiliated Hospital of Gannan Medical University, Ganzhou, China; 2 The School of Basic Medical Sciences, Qilu Medical University, Zibo, China; 3 Longnan Hospital, The First Affiliated Hospital of Gannan Medical University, Longnan, China; 4 The First Clinical Medical College, Gannan Medical University, Ganzhou, China; 5 The School of Basic Medical Sciences, North China University of Science and Technology, Tangshan, China

**Keywords:** adrenocortical carcinoma, anoikis, drug sensitivity, immune microenvironment, Skp2

## Abstract

**Objective:**

Adrenocortical carcinoma (ACC) is a rare but highly aggressive malignancy with limited treatment options. Anoikis plays a critical role in the progression of various cancers; however, its function in ACC remains unclear.

**Methods:**

Based on the GSE19776 and GSE12368 datasets, differentially expressed genes and significantly dysregulated anoikis-related genes were identified. Core ARGs were selected via protein-protein interaction network analysis and least absolute shrinkage and selection operator (LASSO)-Cox regression analysis to construct a risk-scoring model. Functional enrichment analyses, immune microenvironment association analysis, drug sensitivity analysis, mutation profiling, and *in vitro* experiments targeting *SKP2* under routine adherent culture conditions were subsequently performed.

**Results:**

We identified 32 significantly dysregulated ARGs and established a risk model based on six core genes: *BIRC5*, *CASP9*, *CDK1*, *EZH2*, *MDM2*, and *SKP2*. Patients in the high-risk group had significantly shorter overall survival Functional analyses revealed that high-risk Adrenocortical carcinoma was primarily associated with biological processes such as DNA replication and cell cycle regulation. High-risk patients were also characterized by lower immune scores and altered immune cell infiltration patterns. High-risk patients showed increased sensitivity to chemotherapeutic agents including Gallibiscoquinazole, Cisplatin, Axitinib, and Zoledronate. Mutation profiling further identified TP53 missense mutation as a dominant molecular feature. Under routine adherent culture conditions, *SKP2* knockdown inhibited ACC cell proliferation and migration, promoted apoptosis, and induced cell cycle arrest at the G0/G1 phase.

**Conclusion:**

The anoikis-based risk model may serve as a useful prognostic tool for ACC. ARGs may contribute to ACC progression and are associated with immune microenvironment features, providing a potential basis for further mechanistic and translational studies.

## Graphical Abstract

**Figure:**
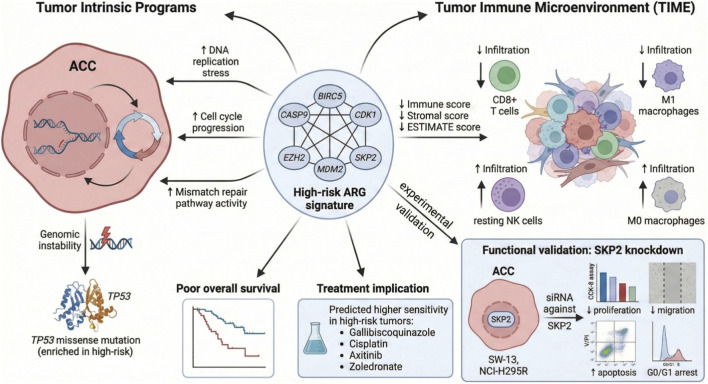

## 1 Introduction

Adrenocortical carcinoma (ACC) is a rare yet highly aggressive endocrine malignancy characterized by rapid progression, limited treatment options, and a poor prognosis. Although surgical resection remains the primary treatment, approximately 30%–40% of patients present with metastatic disease at diagnosis, and the postoperative recurrence rate is as high as 70%, with a 5-year OS rate of less than 30% (Baur et al., 2017; Tran et al., 2016). Current prognostic assessment of ACC relies primarily on clinical staging and histopathological grading, but significant intertumoral heterogeneity limits its accuracy. The predictive power of existing molecular markers, such as *TP53* and *CTNNB1* (Libè et al., 2007; Zheng et al., 2016), is limited, highlighting an urgent need for novel biomarkers to improve risk stratification. Furthermore, the molecular heterogeneity of ACC complicates clinical management, necessitating a deeper understanding of the genetic and immune mechanisms driving tumor progression and therapeutic resistance.

Anoikis is a form of programmed cell death triggered by cell detachment from the extracellular matrix (ECM) or loss of anchorage dependence, playing a critical role in maintaining tissue homeostasis and inhibiting metastasis (Zhang et al., 2021). Recent studies have elucidated molecular pathways regulating anoikis resistance, including cell adhesion molecules, growth factors, and signaling cascades inducing epithelial-mesenchymal transition (EMT) (Adeshakin, 2021). Key downstream mediators such as focal adhesion kinase (FAK) (Alanko and Ivaska, 2016), Src kinase (Li et al., 2019), mitogen-activated protein kinase (MAPK) (Mason et al., 2016), ERK1/2 (Song et al., 2021), Bcl-2 family proteins (Sharma et al., 2021), PI3K/Akt (Moroe t al., 2009; Wang et al., 2022), and insulin-like growth factor receptor (IGF-1R) (Luey and May, 2016) have been demonstrated to promote survival and anti-apoptotic signaling. In breast cancer, anoikis resistance is closely associated with EMT, whereby cells upregulate signaling pathways to evade anoikis and facilitate metastasis (D'Amato et al., 2015; Novikov et al., 2016). In colorectal cancer, the expression profile of ARGs is significantly correlated with patient prognosis, and ARG-based risk models can effectively predict survival outcomes (Zhao et al., 2021). Similarly, IQGAP1 enhances anoikis resistance and metastatic potential in liver cancer by activating the Src/FAK pathway (Mo et al., 2021), while SPIB promotes anoikis resistance in lung cancer via the autolysosomal process (Zhang et al., 2020). In gastric cancer, TCF7L2 transcriptionally activates *PLAUR* to drive anoikis resistance and metastasis, suggesting the existence of both common and cancer-specific mechanisms (Zhang et al., 2022). These findings underscore the critical role of anoikis in cancer metastasis and progression. However, the involvement of ARGs in ACC remains unexplored. Although ACC metastasis is associated with stromal remodeling, no study has yet systematically investigated whether an ARG signature can be used for prognostic stratification or whether it influences ACC progression via immune infiltration and drug sensitivity. This knowledge gap hinders the development of therapeutic strategies targeting anoikis.

To address this gap, this study aimed to systematically construct and validate a robust prognostic signature for ACC based on ARGs. Our research strategy included: a comprehensive bioinformatic analysis of public transcriptomic datasets to identify differentially expressed ARGs and develop a prognostic model; elucidation of the model’s correlations with the tumor immune microenvironment, mutational landscape, and chemotherapeutic response. Furthermore, key findings were validated through *in vitro* experiments. In summary, our results suggest that the ARG signature may serve as a prognostic biomarker associated with ACC progression and immune microenvironment features, providing a potential basis for risk stratification and future mechanistic studies.

## 2 Materials and methods

### 2.1 Data processing

The gene symbols of 434 ARGs were obtained from the study by Chen et al. (2021). Gene expression profiles from the GSE19776 (Demeure et al., 2013) and GSE12368 (Soon et al., 2009) datasets were used to identify DEGs using the “limma” R package. The TCGA-ACC cohort, which contained RNA-seq data with available overall survival information, was used as the training cohort for prognostic model construction, whereas GSE19750 (Legendre et al., 2016) and GSE33371 (Heaton et al., 2012), both based on expression profiling by array, were used as external validation cohorts. Detailed information on all datasets used in this study is summarized in Table 1. For the GEO datasets, background correction, normalization, log2 transformation, and batch correction were performed before downstream analysis, and normalized expression values were used for subsequent model construction and validation. DEGs were defined as those with *P* < 0.05 and log2 |fold change| > 1. Volcano plots and heatmaps were generated using the “ggplot2″ and “pheatmap” packages, respectively.

**TABLE 1:** Summary of datasets used in this study.

Dataset	Database	Sample composition	Data type	Platform	Usage
GSE19776	GEO	59 ACC/4 normal	Microarray	GPL570	DEG analysis
GSE12368	GEO	12 ACC/6 normal	Microarray	GPL570	DEG analysis
TCGA-ACC	TCGA	79 ACC	RNA-seq	Illumina RNA-seq	Training cohort
GSE19750	GEO	44 ACC/4 normal	Microarray	GPL570	Validation cohort
GSE33371	GEO	33 ACC/10 normal	Microarray	GPL570	Validation cohort

Only ACC, samples with available survival information were included in the training and validation analyses where applicable.

### 2.2 Identification of hub genes

PPI data of DEGs were retrieved from the STRING database (Szklarczyk et al., 2019). The PPI network was constructed and visualized using Cytoscape 3.9.1. Candidate hub genes were identified by integrating four distinct analytical methods: the MCODE clustering algorithm, betweenness centrality, degree centrality, and Maximal Clique Centrality (MCC). The final hub genes were determined based on the intersection of results from these four methods.

### 2.3 Prognostic model construction and validation

The LASSO-Cox regression analysis was performed using the “glmnet” package in R. The optimal λ value was determined using cross-validation implemented in the glmnet package (10-fold cross-validation), based on the minimum partial likelihood deviance criterion. Based on OS data from the TCGA-ACC training cohort, six candidate genes and their corresponding coefficients were identified. The risk score for each patient was calculated as follows: Risk Score = ∑Coefgenei × Expgenei. Feature selection and model construction were performed exclusively in the TCGA-ACC training cohort to avoid potential data leakage. Patients in the training cohort were divided into high-risk and low-risk groups based on the median risk score of the training cohort. Kaplan-Meier (KM) survival curves were plotted to compare OS between the two groups, and receiver operating characteristic (ROC) curves were generated using the “survivalROC” package to evaluate the predictive performance of the model. For external validation, the regression coefficients derived from the training cohort were directly applied to the validation cohorts (GSE19750 and GSE33371) to calculate risk scores. Patients in each validation cohort were then stratified into high-risk and low-risk groups using the median risk score within each respective cohort, and KM survival analysis and ROC curve analysis were performed accordingly.

Subsequently, a nomogram integrating the risk score and clinical variables was constructed for the training cohort using the “rms” package to predict 1-, 3-, and 5-year OS probabilities. The predictive performance of the nomogram was evaluated primarily by calibration curves in both the training and validation cohorts.

### 2.4 GO enrichment, KEGG pathway and GSVA analysis

Genes most significantly correlated with the risk score were screened using Pearson correlation analysis with thresholds of (|*r*| > 0.6) and (*P* < 0.05). Gene Ontology (GO) functional enrichment and Kyoto Encyclopedia of Genes and Genomes (KEGG) pathway analyses were performed for these candidate genes using the DAVID online platform (https://david.ncifcrf.gov/). The top five enriched terms ranked by ascending *P*-value were visualized using the “ggplot2″ R package.

GSVA was then conducted using the “GSVA” R package to calculate enrichment scores for DNA replication-related functions in each patient. Gene sets corresponding to specific biological functions were obtained from the AmiGO two web portal (http://amigo.geneontology.org). Patient samples were sorted in ascending order of risk score, and a heatmap generated with the “pheatmap” package was used to display the association between risk scores and enrichment scores of DNA replication-related gene sets. Pearson correlation analysis between risk scores and functional enrichment scores was performed in R Studio to calculate the correlation coefficients (*R*) and *P*-value.

### 2.5 Immune score and tumor-infiltrating immune cell analysis

The ESTIMATE algorithm was used to calculate stromal, immune, and ESTIMATE scores for the training cohort (TCGA-ACC) and validation cohorts (GSE19750 and GSE33371). The CIBERSORT online platform (https://cibersort.stanford.edu/) (Newman et al., 2015) was used to estimate the relative proportions of 22 tumor-infiltrating immune cell types based on the LM22 signature matrix, with 1,000 permutations. Normalized gene expression matrices were used as input. All samples were included in the downstream analysis without additional filtering based on the CIBERSORT *P*-value.

### 2.6 Gene mutation and drug sensitivity analysis

In the TCGA-ACC cohort, patients were divided into high-risk and low-risk groups based on the median risk score of the training cohort. Differential mutation analysis between groups was performed using the “maftools” R package, and results were visualized using mutation waterfall plots. In addition, the “oncoPredict” package was used to predict sensitivity scores of the two risk groups to common ACC chemotherapeutic agents, including Gallibiscoquinazole, Cisplatin, Axitinib, and Zoledronate.

### 2.7 Cell culture and transfection

The human ACC cell lines NCI-H295R and SW-13 were purchased from Shanghai Yizefeng Biotechnology Co., Ltd. NCI-H295R cells were cultured in DMEM/F12 medium (Beijing EallBio) supplemented with 10% fetal bovine serum (FBS), and SW-13 cells were maintained in DMEM medium (Beijing EallBio) containing 10% FBS. All cells were routinely cultured at 37 °C in a 5% CO_2_ atmosphere. Unless otherwise indicated, all *in vitro* functional assays were performed under routine adherent culture conditions rather than dedicated anoikis-inducing conditions. Three independent *SKP2*-targeting small interfering RNAs (siRNAs) and a negative control siRNA (si-NC) were synthesized by Shanghai Qianmo Biotechnology Co., Ltd (see Supplementary Table S1 for sequences). Cell transfection was performed using Lipofectamine 2000 transfection reagent (Invitrogen Corporation) according to the manufacturer’s instructions. The siRNA showing the most stable and efficient knockdown based on RT-qPCR and Western blotting was selected for subsequent functional assays.

### 2.8 Western blotting analysis

After transfection, NCI-H295R and SW-13 cells were lysed with RIPA lysis buffer (Beyotime) to extract total protein. Protein concentration was determined using a BCA protein assay kit (Beyotime). Equal amounts of protein (20 μg per sample) were separated by 12% SDS-PAGE (Epizyme) and transferred onto PVDF membranes (Beyotime). The membranes were then blocked with 5% non-fat milk in TBST for 2 h at room temperature. After blocking, the membranes were incubated overnight at 4 °C with primary antibodies against GAPDH and SKP2 (Beyotime). Following three washes with TBST, the membranes were incubated with HRP-conjugated secondary antibodies (Beyotime) for 1 h at room temperature. Protein bands were visualized using an ECL detection kit (Beyotime) and imaged with a ChemiDoc imaging system (Bio-Rad).

### 2.9 RT-qPCR assay

Total RNA was extracted from NCI-H295R and SW-13 cells using the TRIzol method. cDNA was synthesized using a reverse transcription kit (Tianjin Zhongshi Gene Technology Co., Ltd.). RT-qPCR was performed using specific primers synthesized by Invitrogen: human *SKP2* forward primer: 5′-GATGTGACTGGTCGGTTGCTGT-3′, reverse primer: 5′-GAGTTCGATAGGTCCATGTGCTG-3’; human *GAPDH* forward primer: 5′-GTCTCCTCTGACTTCAACAGCG-3′, reverse primer: 5′-ACCACCCTGTTGCTGTAGCCAA-3’. *GAPDH* was used as the internal reference gene. The relative expression level of *SKP2* was calculated using the 2^−ΔΔCT^ method.

### 2.10 Cell proliferation and wound healing assays

Cell proliferation was assessed using a CCK-8 kit (Beijing Dojindo). Transfected NCI-H295R and SW-13 cells were seeded into 96-well plates at a density of 1 × 10^4^ cells per well. After 24 h, the culture medium was aspirated and replaced with 100 μL of fresh medium containing 10% CCK-8 reagent. Following incubation at 37 °C for 1.5 h, the absorbance was measured at 450 nm using a microplate reader (Tecan).

Cell migration ability was evaluated using a wound healing assay. Transfected SW-13 cells were seeded into 48-well plates and cultured until they reached 80%–90% confluence. A uniform wound was created in the monolayer using a sterile 200 μL pipette tip. After washing with PBS to remove detached cells, the medium was replaced with DMEM containing 1% FBS. Wound areas were photographed at 0 h and 24 h using an inverted phase-contrast microscope. The wound area was quantified using ImageJ software, and the migration rate was calculated as follows (1 - wound area at 24 h/wound area at 0 h) × 100 %. All data were obtained from three independent experiments.

### 2.11 Apoptosis and cell cycle analysis

Cell apoptosis was detected using an Annexin V-FITC/PI apoptosis detection kit (Yeasen). Twenty-four hours after transfection, cells were collected and digested with trypsin without EDTA, resuspended in 100 μL of binding buffer, and incubated with 5 μL of Annexin V-FITC and 10 μL of propidium iodide (PI) solution for 15 min at room temperature in the dark. Subsequently, 400 μL of binding buffer was added to each tube, and the samples were analyzed using a FACS Calibur flow cytometer (BD Biosciences). The percentage of apoptotic cells was determined using FlowJo software. All apoptosis experiments were performed in three independent biological replicates.

Cell cycle distribution was analyzed using a cell cycle detection kit (BD Biosciences). Cells were collected 24 h after transfection, washed twice with pre-cooled PBS, and fixed in 95% ethanol at 4 °C for 12–24 h. Fixed cells were stained with a staining buffer containing PI and RNase A. The samples were analyzed using a FACS Calibur flow cytometer with an excitation wavelength of 488 nm for PI red fluorescence. Data were analyzed using FlowJo software. All cell cycle experiments were performed in three independent biological replicates.

### 2.12 Statistical analysis

Primary bioinformatic and statistical analyses were performed using R software (version 4.2.1). These included integration and normalization of mRNA expression data, LASSO-Cox regression analysis, survival analysis, ROC curve analysis, ESTIMATE analysis, and CIBERSORT-based immune cell deconvolution. Pearson correlation analysis was used to assess the correlation between risk scores and DNA replication-related gene sets. Group comparisons for tumor-infiltrating immune cell (TIIC) proportions and chemotherapeutic drug sensitivity were performed using the nonparametric Wilcoxon rank-sum test. Comparisons between two groups were conducted using Student’s t-test, and comparisons among multiple groups were performed using one-way analysis of variance (ANOVA). Experimental data are presented as mean ± SD from at least three independent biological replicates. A *P*-value <0.05 was considered statistically significant.

## 3 Results

### 3.1 DEGs and hub genes in ACC

**FIGURE 1:**
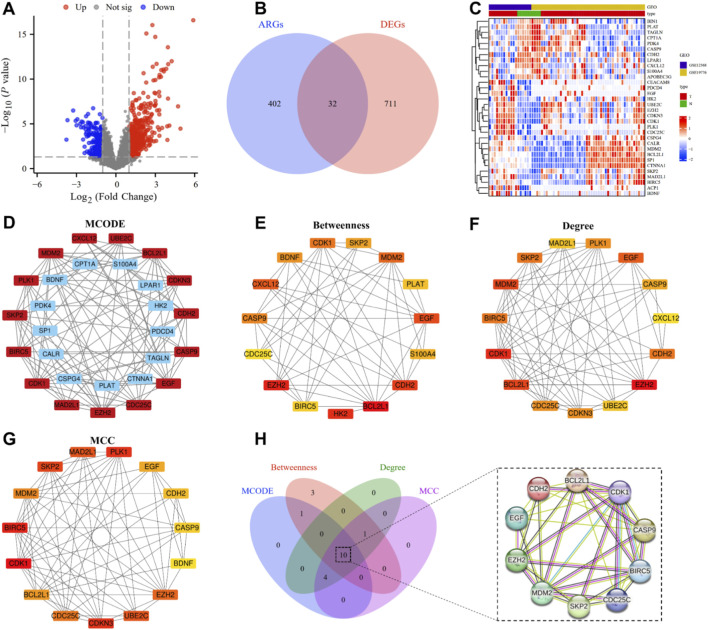
DEGs and hub genes **(A)** Volcano plot of 743 DEGs in GSE19776 and GSE12368 datasets **(B)** Venn diagram showing 32 differentially expressed ARGs **(C)** Heatmap of the 32 differentially expressed ARGs in GSE19776 and GSE12368 **(D)** PPI network of the differentially expressed ARGs and the key module identified by MCODE **(E–G)** Top 15 candidate ARGs ranked by betweenness, degree, and MCC, respectively **(H)** Venn diagram showing 10 hub ARGs identified by the four algorithms.

By analyzing the gene expression profiles from the GSE19776 and GSE12368 datasets (normal adrenal cortex tissue vs. tumor tissue), we screened for differentially expressed ARGs. The volcano plot revealed a total of 743 DEGs across the two datasets (Figure 1A). Among 434 pre-selected ARGs, 32 genes exhibited significant differential expression (Supplementary Table S2). Intersection analysis (Venn diagram, Figure 1B) and hierarchical clustering (heatmap, Figure 1C) collectively confirmed the specific expression pattern of these 32 ARGs.

A PPI network of the 32 DEGs was constructed based on the STRING database and refined using the MCODE plugin in Cytoscape for module analysis (Figure 1D). The top 15 candidate genes were evaluated topologically using betweenness, degree, and MCC algorithms. Ten core genes were ultimately identified: *EZH2*, *CDC25C*, *MDM2*, *SKP2*, *BIRC5*, *CDK1*, *BCL2L1*, *EGF*, *CDH2*, and *CASP9* (Figures 1E–H). These genes were consistently prioritized across all three computational methods.

### 3.2 Construction and validation of the prognostic model

**FIGURE 2:**
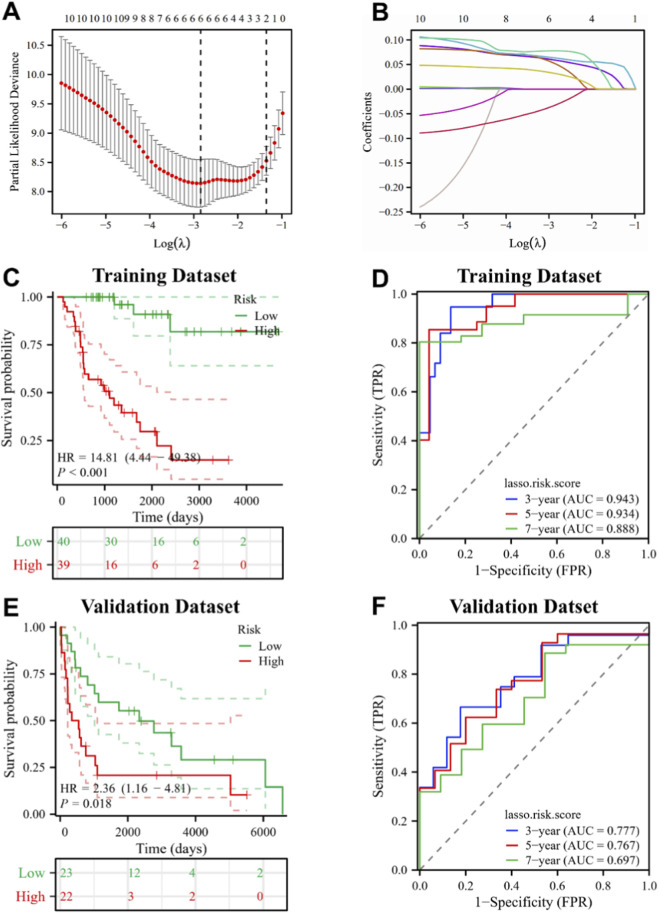
Development and validation of the OS-prediction model for ACC **(A)** Prognostic LASSO coefficient selection **(B)** Prognostic LASSO variable trajectory. Survival curves **(C)** and ROC curves **(D)** for low-risk and high-risk subgroups in the training cohort (TCGA-ACC). Survival curves **(E)** and ROC curves **(F)** for low-risk and high-risk subgroups in the validation cohort (GSE19750 and GSE33371).

We constructed a predictive model for OS using LASSO regression analysis within the TCGA-ACC training cohort. Feature selection identified six prognostic genes (*BIRC5*, *CASP9*, *CDK1*, *EZH2*, *MDM2*, and *SKP2*), with the optimal regularization parameter (λ) determined by cross-validation (Figures 2A,B). The risk score was calculated as follows: Risk Score = (0.059 × *BIRC5* expression) + (0.051 × *CASP9* expression) + (0.063 × *CDK1* expression) + (0.078 × *EZH2* expression) + (−0.032 × *MDM2* expression) + (0.031 × *SKP2* expression). Patients in the training cohort were divided into low-risk and high-risk groups according to the median risk score of the training cohort. Kaplan-Meier analysis revealed significantly poorer OS in the high-risk group (*P* < 0.001, Figure 2C). Time-dependent ROC curves demonstrated good predictive accuracy in the training cohort, with AUC values of 0.943 (3-year), 0.934 (5-year), and 0.888 (7-year) (Figure 2D). For external validation, risk scores were calculated in GSE19750 and GSE33371 using the coefficients derived from the training cohort, and patients in each validation cohort were stratified according to the median risk score within that respective cohort. Patients in the high-risk group had shorter OS (*P* = 0.018, Figure 2E), and the predictive performance remained moderate and acceptable (AUC values: 3-year 0.777, 5-year 0.767, 7-year 0.697, Figure 2F). KM analysis of the six prognostic ARGs within the TCGA-ACC training cohort showed that five ARGs (*BIRC5*, *CASP9*, *CDK1*, *EZH2*, and *SKP2*) were significantly associated with OS (*P* < 0.001, Supplementary Figure S1). Univariate and multivariate Cox regression analyses assessing the prognostic value of the risk score and clinical parameters revealed that the risk score was an independent prognostic factor for OS in both the training cohort (Supplementary Figures S2A,B) and the validation cohorts Supplementary Figures S2C,D).

### 3.3 Association of the risk score with clinicopathological features

**FIGURE 3:**
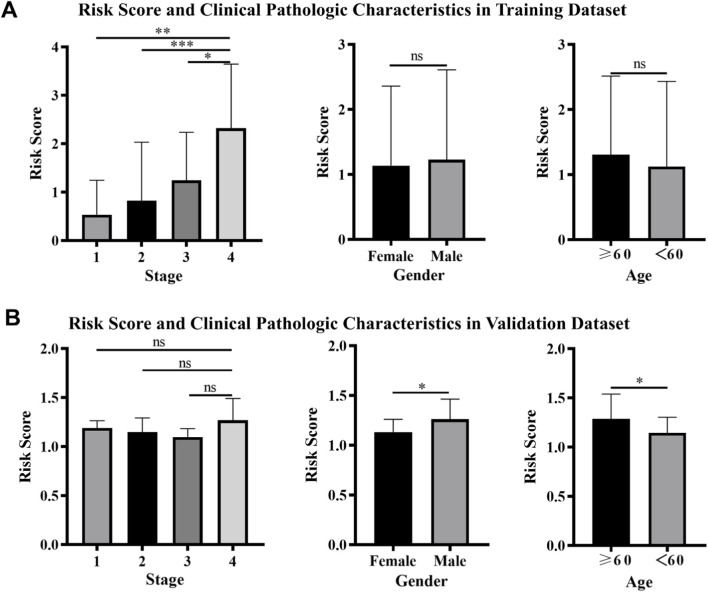
Association between risk score and clinicopathological characteristics of ACC **(A,B)** Bar charts showing the distribution of risk scores across clinicopathological subgroups in the training and validation cohorts. Data are presented as mean ± SD; *P < 0.05; **P < 0.01; ***P < 0.001; ns, not significant.

Patients in different risk groups exhibited distinct patterns of clinicopathological feature distribution. In both the training and validation sets, increasing risk scores showed an asymmetric distribution with patient outcome, tumor stage, sex, age, and the expression levels of *BIRC5*, *CASP9*, *CDK1*, *EZH2*, *MDM2*, and *SKP2* (Supplementary Figures S3A,B). In the training cohort, patients with stage IV tumors had significantly higher risk scores than those with stage III, II, and I tumors (*P* < 0.05) (Figure 3A). In the validation cohorts, male patients had significantly higher risk scores than females (*P* < 0.05), and patients aged ≥60 years had higher risk scores than those <60 years (*P* < 0.05) (Figure 3B).

### 3.4 Close association of the risk score with DNA replication and cell cycle

**FIGURE 4:**
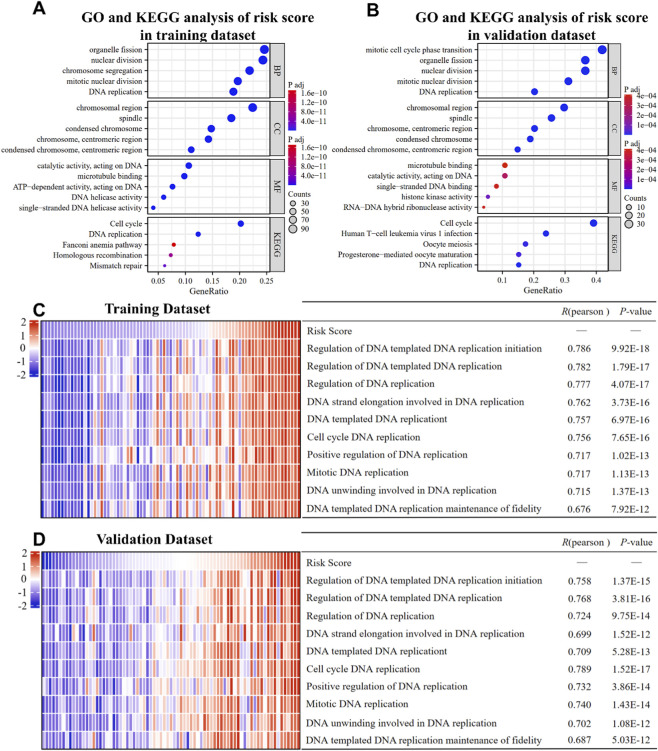
Function enrichment analysis **(A,B)** GO and KEGG analyses of biological processes and pathways associated with the risk score in the training and validation cohorts **(C,D)** GSVA analysis of risk scores and DNA replication-related gene sets in the training and validation cohorts.

To explore the biological significance of the risk score, we performed GO enrichment, KEGG pathway, and GSVA analyses. Consistently across both the training and validation cohorts, the risk score showed significant enrichment in cell cycle-related biological processes (DNA replication, organelle fission, nuclear division), chromosomal structural components (chromosomal region, spindle apparatus, condensed chromosome), and DNA-interacting molecular functions (microtubule binding, DNA-dependent catalytic activity, single-stranded DNA binding) (Figures 4A,B). KEGG pathway analysis further confirmed the involvement of three core pathways: DNA replication, cell cycle regulation, and mismatch repair (Figures 4A,B).

GSVA of DNA replication-related gene sets in ACC, performed on both the training and validation cohorts, revealed a stepwise increase in functional enrichment scores with rising risk scores (Figures 4C,D). The risk score showed a significant positive correlation with key DNA replication pathways, including cell cycle DNA replication, regulation of DNA-templated replication initiation, and regulation of DNA-templated replication processes (Figures 4C,D).

### 3.5 The individualized prediction model showed favorable calibration performance

**FIGURE 5:**
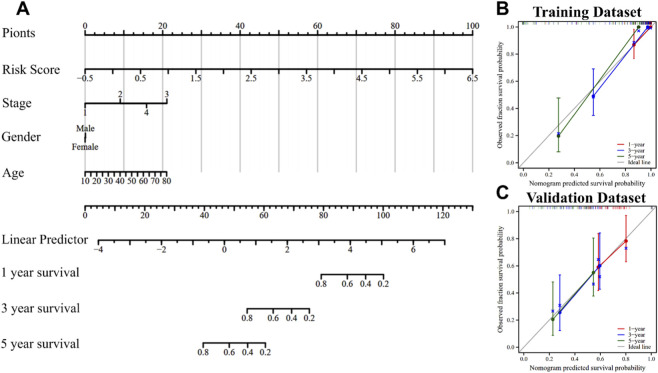
Development and validation of the ACC prognostic nomogram **(A)** Nomogram for estimating 1-, 3-, and 5-year OS probabilities **(B,C)** Calibration curves showing the agreement between predicted and observed survival probabilities in the training and validation cohorts.

To facilitate prognostic assessment in ACC, we developed an individualized prediction model integrating the risk score and clinical variables, including tumor stage, sex, and age. As shown in Figure 5A, the nomogram can estimate the 1-, 3-, and 5-year OS probabilities of ACC patients. The calibration curves showed acceptable agreement between predicted and observed survival probabilities in the training and validation cohorts (Figures 5B,C), suggesting that the nomogram may serve as a potentially useful integrated prognostic tool. However, its clinical utility still requires further evaluation in larger prospective cohorts.

### 3.6 Differences in immune infiltration between risk groups

**FIGURE 6:**
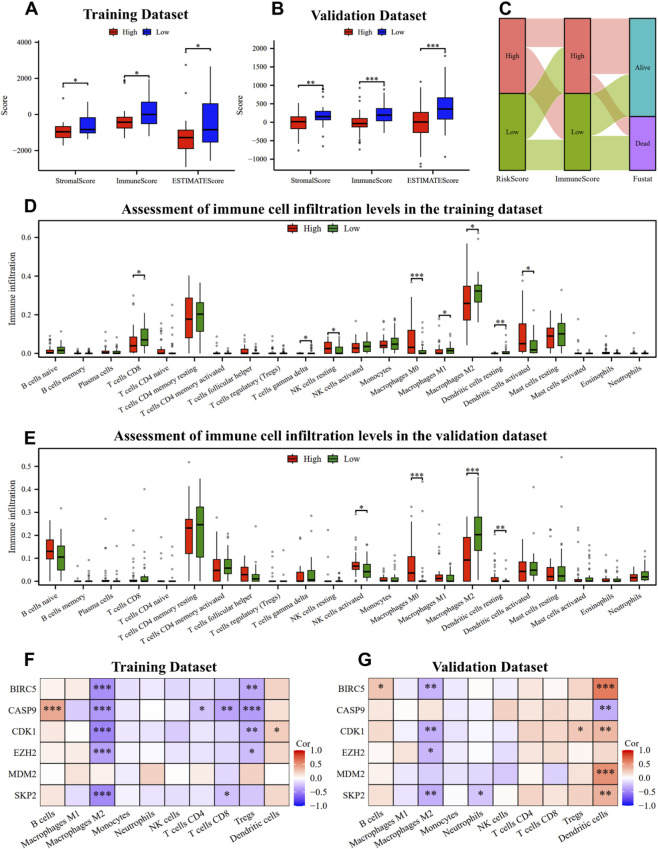
Immune infiltration between risk groups. Comparisons of stromal score, immune score, and ESTIMATE score between high- and low-risk groups in the training **(A)** and validation cohort **(B,C)** Sankey diagram illustrating the associations among risk score, immune score, and survival status. Box plots comparing proportions of TIICs between low- and high-risk groups in the training cohort **(D)** and validation cohort **(E)**. Correlation heatmaps between prognosis-associated ARGs and TIICs in the training cohort **(F)** and validation cohort **(G)**. Data are presented as mean ± SD; *P < 0.05; **P < 0.01; ***P < 0.001.

Given the critical role of the tumor microenvironment (TME) in malignant progression, we used the ESTIMATE and CIBERSORT algorithms to assess differences in immune cell infiltration between risk groups. ESTIMATE analysis revealed that in both the training and validation cohorts, the high-risk group had significantly lower immune, stromal, and ESTIMATE scores than the low-risk group (Figures 6A,B). Lower immune scores were also associated with poorer survival rates (Figure 6C). Comparative analysis of TIICs showed that, in the training cohort, the high-risk group exhibited significantly reduced infiltration of CD8^+^ T cells, M1 macrophages, and M2 macrophages, but increased resting NK cells and M0 macrophages compared with the low-risk group (Figure 6D). In the validation cohort, the high-risk group showed significantly reduced M2 macrophage infiltration, together with increased M0 macrophages and resting dendritic cells (Figure 6E). Furthermore, the six OS-related ARGs demonstrated subgroup-specific correlations with particular TIIC subsets in both the training and validation cohorts (Figures 6F,G). Overall, these findings suggest that the risk model is associated with immune microenvironment features, although the immune infiltration patterns were not fully consistent across datasets.

### 3.7 Molecular features of TP53 missense mutation and drug sensitivity analysis in high-risk ACC

**FIGURE 7:**
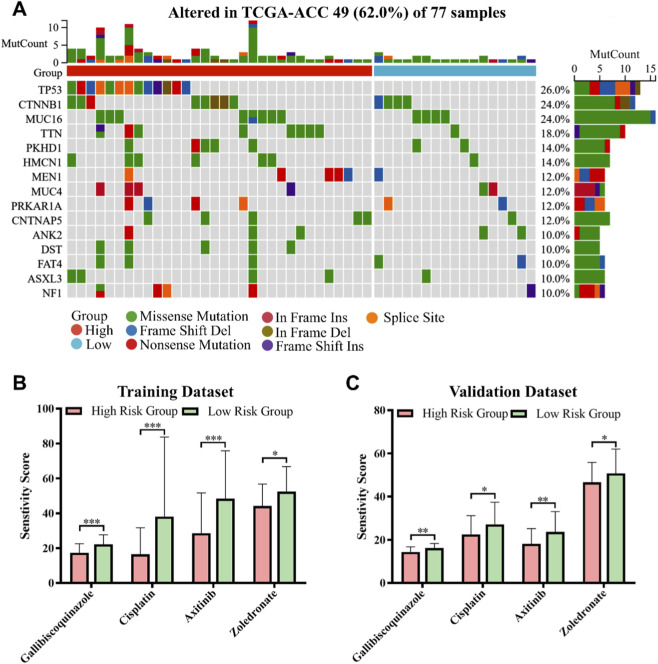
Mutation landscape and drug sensitivity analysis **(A)** Mutation waterfall plot of TCGA-ACC patients stratified by risk score. Drug sensitivity profiling of ACC stratified by risk groups (high-risk vs. low-risk) **(B)** training cohort **(C)** validation cohort. All data are presented as mean ± SD; *P < 0.05; **P < 0.01; ***P < 0.001.

The results of the mutational landscape analysis are shown in Figure 7A. Whole-exome sequencing revealed significant intergroup heterogeneity in the TCGA-ACC cohort, with the high-risk and low-risk groups presenting distinct variant profiles. Missense mutations were the most predominant variant type, followed by nonsense mutations and splice site variants. The *TP53* gene had the highest mutation frequency (26%), followed by *CTNNB1* (24%) and *MUC16* (24%). Notably, the incidence of *TP53* mutations was significantly higher in high-risk tumors compared to low-risk tumors. Based on this risk stratification, we further predicted the sensitivity of the high-risk and low-risk groups to various chemotherapeutic agents by analyzing anticancer drug sensitivity data. The results showed that in both the training (Figure 7B) and validation (Figure 7C) cohorts of ACC patients, the high-risk group exhibited lower drug sensitivity scores (indicating a potentially higher therapeutic response) for Gallibiscoquinazole, Cisplatin, Axitinib, and Zoledronate, suggesting that high-risk patients might be more responsive to these drugs.

### 3.8 Knockdown of *SKP2* inhibits the malignant progression of ACC cells

**FIGURE 8:**
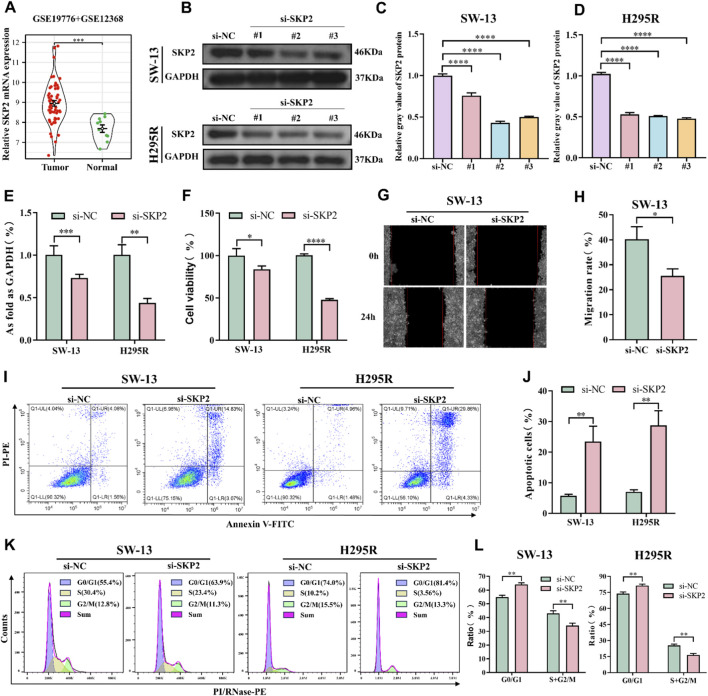
Functional effects of *SKP2* knockdown in ACC cells under routine adherent culture conditions **(A)** *SKP2* expression in normal adrenal tissues and ACC (GSE19776+GSE12368) **(B–E)** Knockdown efficiencies of three independent *SKP2*-targeting siRNAs were evaluated at the protein and mRNA levels, and the siRNA with the strongest and most stable knockdown was used for the subsequent functional assays **(F)** Cell proliferation was assessed by CCK-8 assay **(G,H)** Cell migration ability was evaluated by wound healing assay **(I,J)** Cell apoptosis was analyzed by flow cytometry using Annexin V-FITC/PI staining **(K,L)** Cell cycle distribution was analyzed by flow cytometry after PI staining. Data are representative of three independent experiments (n = 3 biological replicates; mean ± SD; *P < 0.05; **P < 0.01; ***P < 0.001; ****P < 0.0001).

Although *SKP2* has been identified as a gene associated with ACC prognosis, its specific functional role in ACC remains unclear. Therefore, this study investigated the impact of *SKP2* on ACC cell behavior *in vitro* under routine adherent culture conditions. We initially screened three independent *SKP2*-targeting siRNAs and used si-NC as the negative control. Based on RT-qPCR and Western blotting, the siRNA showing the most stable and efficient knockdown was selected for subsequent functional assays. Compared with normal adrenal tissues, tumor tissues exhibited higher mRNA expression levels of *SKP2* (Figure 8A). The knockdown efficiency was confirmed by Western blotting and RT-qPCR (Figures 8B–E). CCK-8 assay results demonstrated that *SKP2* knockdown significantly inhibited the proliferation ability of both SW-13 and H295R cells compared with the si-NC group (Figure 8F). Wound healing assays further showed that *SKP2* knockdown significantly attenuated the migratory capacity of SW-13 cells (Figures 8G,H). Apoptosis assays revealed that *SKP2* knockdown led to a significant increase in the proportion of apoptotic SW-13 and H295R cells (Figures 8I,J). Additionally, cell cycle analysis indicated that *SKP2* knockdown resulted in a significant increase in the proportion of cells in the G0/G1 phase and a decrease in the proportion of cells in the S phase in both SW-13 and H295R cells (Figures 8K,L). In summary, *SKP2* knockdown was associated with reduced proliferation and migration, increased apoptosis, and G0/G1 phase arrest in ACC cells under routine adherent culture conditions.

## 4 Discussion

ACC remains a therapeutic challenge due to its aggressive nature, limited treatment options, and the lack of reliable prognostic biomarkers. Anoikis plays an important role in cancer progression and has attracted increasing attention in recent years (Sattari Fard et al., 2022; Thuong et al., 2024). However, the association between ARGs and ACC has not been fully explored. In this study, we integrated genomic, immune, and clinical data to construct a prognostic model and to explore its biological and clinical associations. Our findings suggest that ARGs may have prognostic relevance in ACC and may be linked to key pathways involved in ACC progression.

The identification of 32 differentially expressed ARGs, including hub genes such as *BIRC5*, *CASP9*, and *EZH2*, suggests that dysregulation of anoikis-related pathways may participate in ACC pathogenesis. These genes are functionally linked to apoptotic escape, cell cycle progression, and genomic instability, which are hallmarks of aggressive tumors. For instance, *BIRC5* (survivin) is a well-established anti-apoptotic protein overexpressed in multiple malignancies and associated with chemoresistance and poor prognosis (Siragusa et al., 2024). *EZH2* has also been implicated in tumorigenesis through transcriptional reprogramming and silencing of tumor suppressor genes (Behrens et al., 2013; Chase and Cross, 2011; Xu et al., 2013). The prognostic model based on six ARGs showed good discrimination in the training and validation cohorts, although the decrease in AUC values in the validation cohorts suggests a potential risk of overfitting and indicates that further validation in larger independent datasets is warranted. In addition, *MDM2* was the only gene with a negative coefficient in the risk formula. This result should be interpreted cautiously, as coefficients in a multigene LASSO-Cox model reflect contributions within a multivariable context and may be influenced by collinearity, cohort-specific molecular features, and the complex interaction between *MDM2* and *TP53* signaling, rather than implying a protective role of *MDM2* in ACC.

Functional enrichment analysis revealed that the high-risk ACC patient group is characterized by aberrant activation of DNA replication and cell cycle-related pathways. The strong correlation between the risk score and DNA replication-related gene sets (e.g., cell cycle DNA replication, regulation of DNA-templated replication) suggests that dysregulated replication stress and genomic instability may be key drivers of ACC progression. This finding is further supported by the predominance of *TP53* missense mutations in high-risk tumors. *TP53* mutations, which impair DNA damage repair and promote uncontrolled proliferation, are well-documented mechanisms in ACC and other cancers (Marei et al., 2021; Baslan et al., 2022). The co-occurrence of *CTNNB1* and *MUC16* mutations in high-risk tumors highlights additional pathogenic pathways such as Wnt signaling activation and immune evasion (Lefèvre et al., 2015; Doghman et al., 2008; Ruggiero et al., 2023), underscoring the complexity of ACC’s molecular mechanisms.

The tumor immune microenvironment (TIME) is a dynamic network composed of immune cells, immunomodulatory molecules, and their interactions within tumor tissue, and it plays an important role in tumor progression and treatment response (Surendran et al., 2021). In the present study, ESTIMATE analysis showed that high-risk ACC patients had significantly lower stromal and immune scores than the low-risk group. CIBERSORT analysis further suggested associations between the risk score and several immune cell subsets, including macrophage populations, CD8^+^ T cells, and resting NK cells. Altered abundances of these cellular subsets have been widely confirmed to be associated with poor prognosis in malignancies (He et al., 2020). However, the immune infiltration patterns were not fully consistent between the training and validation cohorts, with macrophage-related changes showing relatively more consistent trends than other cell types. Therefore, these findings should be interpreted as associations between the risk model and immune microenvironment features rather than evidence that the model can directly predict response to immunotherapy. Because our immune analyses were based on retrospective transcriptomic deconvolution rather than immunotherapy-treated ACC cohorts, the potential implication of this model for immunotherapy response remains to be prospectively validated.

Drug sensitivity analysis further supports the potential clinical relevance of risk stratification. In this study, high-risk ACC patients showed increased predicted sensitivity to Gallibiscoquinazole, Cisplatin, Axitinib, and Zoledronate. However, predicted drug sensitivity does not necessarily translate into improved clinical outcomes, and these observations should be interpreted cautiously until supported by experimental and clinical validation.

Among these prognosis-related ARGs, the roles and molecular mechanisms of *BIRC5*, *CDK1*, *EZH2*, *CASP9*, and *MDM2* in *ACC* have been reported in the literature, whereas the function of *SKP2* in ACC remains less well characterized (Gao et al., 2023; Ren et al., 2022; Drelon et al., 2016; Bussey et al., 2016; Subramanian et al., 2023). For this reason, *SKP2* was selected for preliminary functional validation. Under routine adherent culture conditions, *SKP2* knockdown inhibited ACC cell proliferation and migration, induced cell cycle arrest, and promoted apoptosis *in vitro*, suggesting that *SKP2* participates in malignant phenotypes of ACC cells. However, because these assays were not performed under dedicated anoikis-inducing conditions, they should not be interpreted as direct evidence that *SKP2* regulates anoikis resistance in ACC. In addition, only *SKP2* was experimentally evaluated, and the remaining genes in the model warrant further functional investigation.

However, this study has several limitations. First, although the prognostic model showed promising performance, the limited sample size of ACC and the higher predictive accuracy in the training cohort compared with the validation cohorts indicate that potential overfitting cannot be completely excluded. Second, the use of cohort-specific median cutoffs may limit direct comparability across datasets. Third, the immune analyses were based on ESTIMATE and CIBERSORT deconvolution of retrospective transcriptomic data, and no additional filtering based on the CIBERSORT P-value was applied, which may affect the robustness of immune cell estimation. Fourth, the *in vitro* assays were performed under routine adherent conditions rather than dedicated anoikis-inducing conditions. Fifth, although three independent SKP2-targeting siRNAs were initially screened, the subsequent functional assays were performed using only the siRNA with the highest knockdown efficiency, and potential off-target effects therefore cannot be fully excluded. Finally, no *in vivo* experiments were performed. Future studies using larger multicenter cohorts, additional functional assays, rescue experiments, and animal models are needed to further validate these findings.

## 5 Conclusion

This study suggests that an anoikis-related gene signature may serve as a potentially useful prognostic tool in ACC and is associated with DNA replication-related pathways, immune microenvironment features, and drug sensitivity patterns. The nomogram may provide a potentially useful integrated approach for risk assessment, although its clinical utility requires further validation. *SKP2* emerged as a candidate gene for further investigation, but additional mechanistic studies, independent cohort validation, and *in vivo* experiments are needed before definitive translational conclusions can be drawn.

## Data Availability

Publicly available datasets were analyzed in this study. This data can be found here: https://portal.gdc.cancer.gov/projects/TCGA-ACC; https://www.ncbi.nlm.nih.gov/geo/query/acc.cgi?acc=GSE19776; https://www.ncbi.nlm.nih.gov/geo/query/acc.cgi?acc=GSE12368; https://www.ncbi.nlm.nih.gov/geo/query/acc.cgi?acc=GSE19750; https://www.ncbi.nlm.nih.gov/geo/query/acc.cgi?acc=GSE33371.
