# Supplementary material for: An anoikis-based risk model predicts outcomes and is associated with the immune microenvironment in adrenocortical carcinoma
Source: Front Mol Biosci. 2026 Jun 11;13:1779180. doi: 10.3389/fmolb.2026.1779180 (PMC13293581; doi:10.3389/fmolb.2026.1779180)
Supplement: Supplementary file 1 [file DataSheet1.docx]

Supplementary Material

# Supplementary Figures


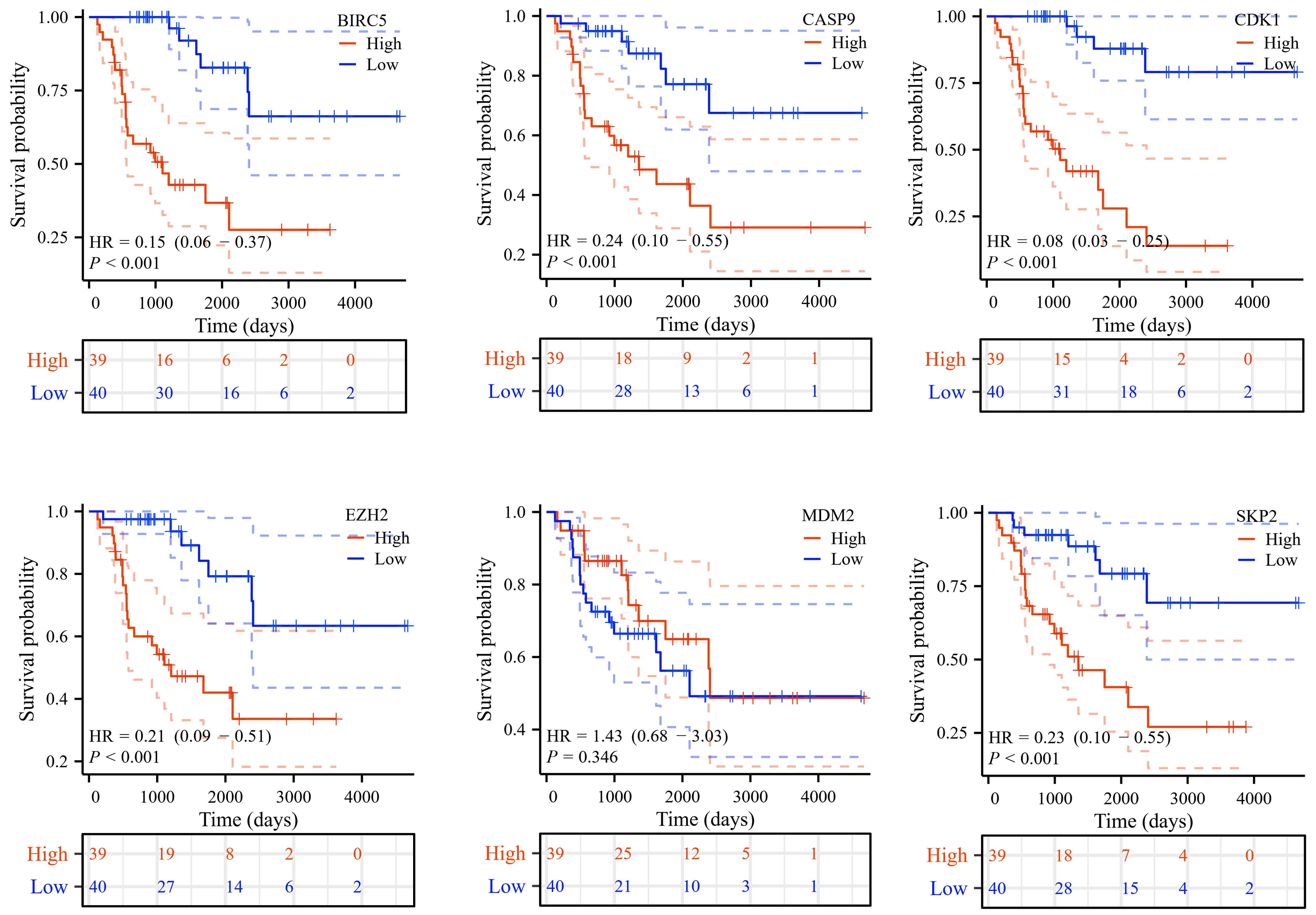


**Supplementary Figure 1.** Kaplan-Meier analysis of differentially expressed ARGs for OS in TCGA-ACC.


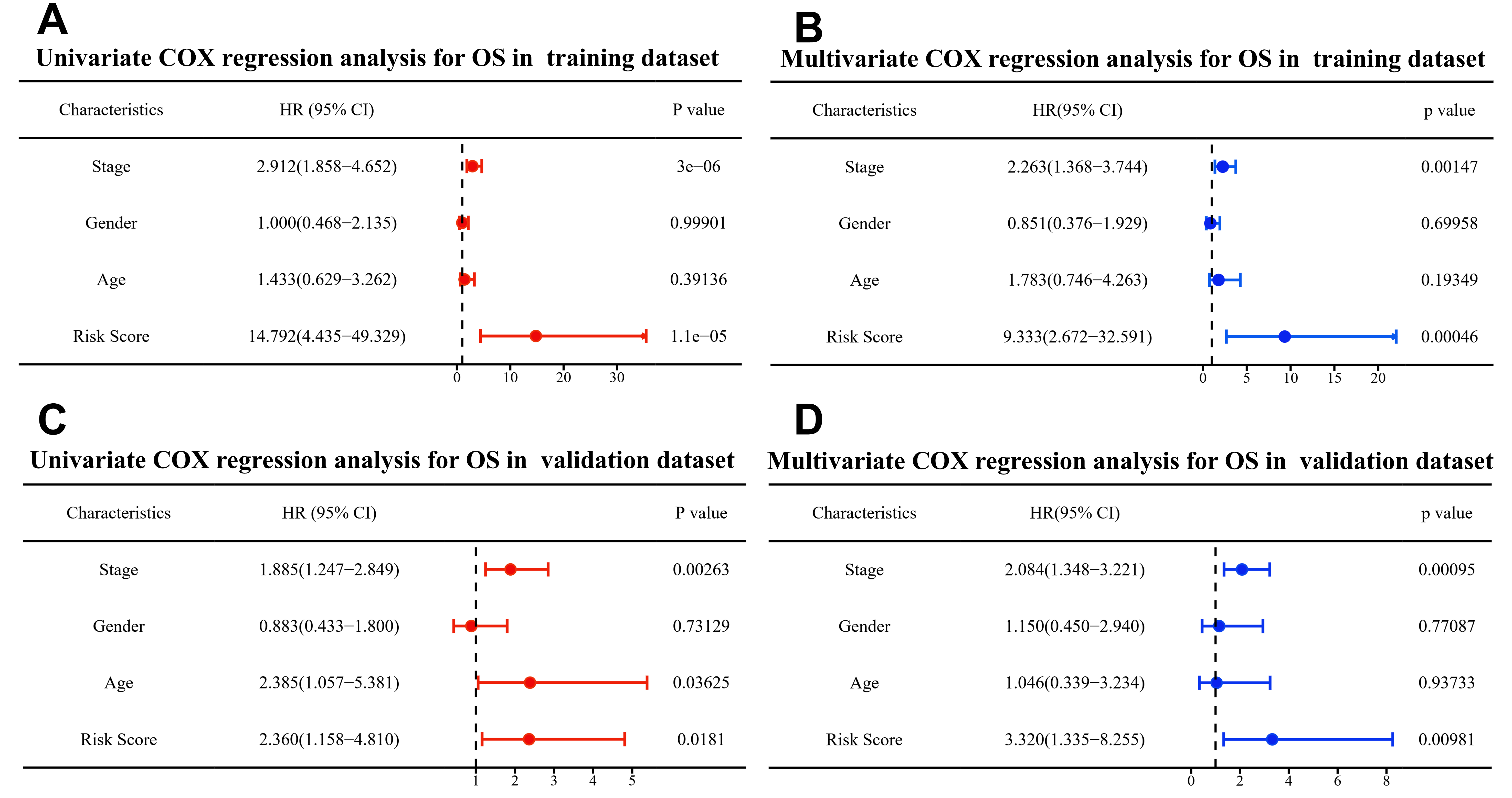


**Supplementary Figure 2.** Univariate and multivariate Cox regression analyses. Univariate (**A**) and multivariate (**B**) Cox regression analyses in the training cohort. Univariate (**C**) and multivariate (**D**) Cox regression analyses in the validation cohort.


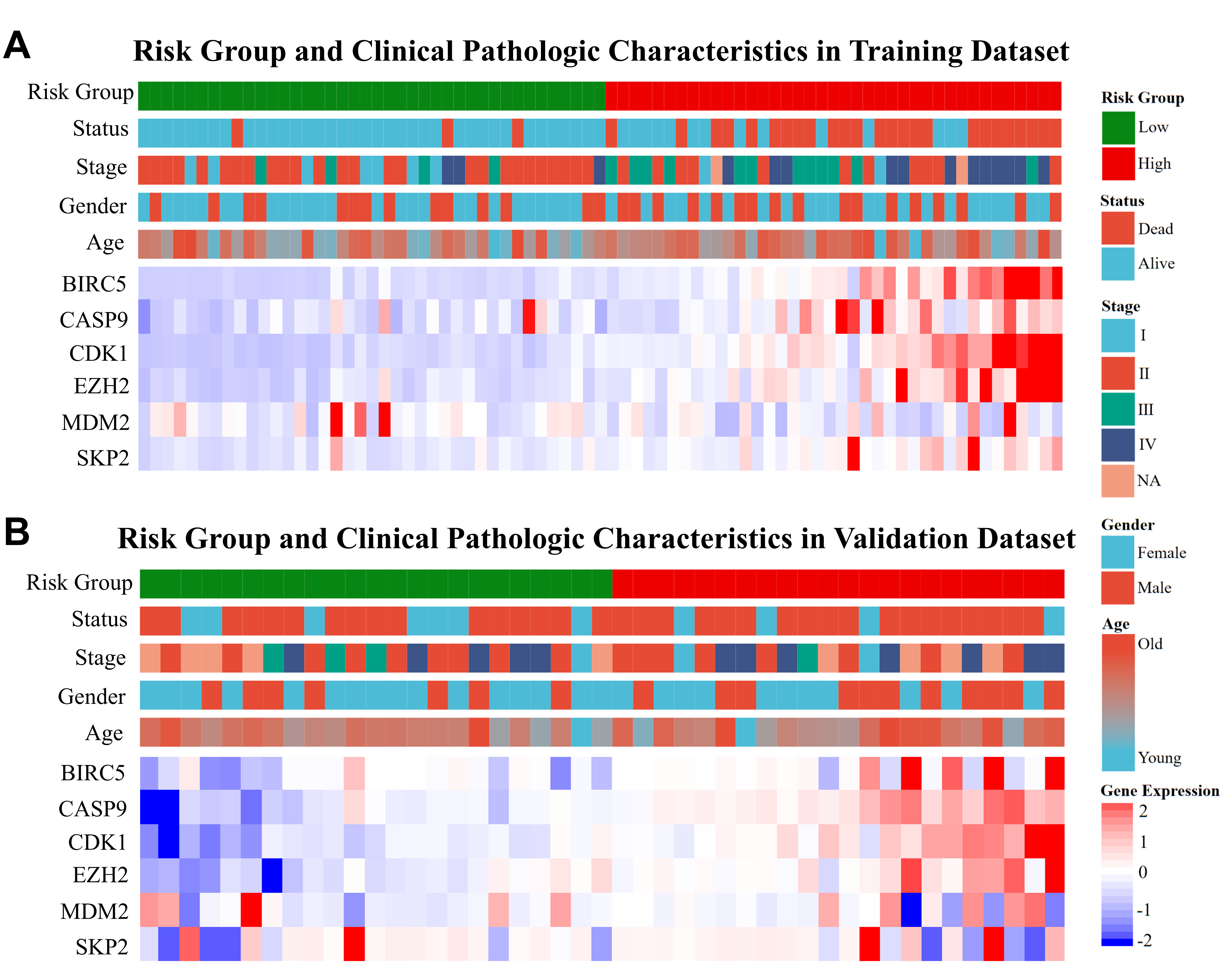


**Supplementary Figure 3.** Association between risk score and clinicopathological characteristics of ACC. (**A, B**) The heatmap showed the clinical-pathologic factors and 6 representative genes for each ACC in ascending order of the recurrence score in training and validation groups.

# Supplementary Tables

**Supplementary Table 1.** The sequences of primer and siRNA oligonucleotides.

| **siRNA** | **Target sequence (5’-3’)** | **Senese (5’-3’)** | **Antisense (5’-3’)** |
| --- | --- | --- | --- |
| Negative control | TTCTCCGAACGTGTCACGT | UUCUCCGAACGUGUCACGU | ACGUGACACGUUCGGAGAA |
| si-SKP2-1 | GTGATAGTGTCATGCTAAA | GUGAUAGUGUCAUGCUAAA | UUUAGCAUGACACUAUCAC |
| si-SKP2-2 | CAAATTTAGTGCGACTTAA | CAAAUUUAGUGCGACUUAA | UUAAGUCGCACUAAAUUUG |
| si-SKP2-3 | GGCTGTTGCGCATGTGTCA | GGCUGUUGCGCAUGUGUCA | UGACACAUGCGCAACAGCC |

**Supplementary Table 2.** Differentially expressed ARGs.

| **Gene** | **logFC** | **P Value** |
| --- | --- | --- |
| TAGLN | 3.30597 | 8.86E-07 |
| CDK1 | -3.24938 | 3.27E-07 |
| CDKN3 | -2.92871 | 3.06E-06 |
| PLAT | 2.716841 | 9.42E-07 |
| CDH2 | 2.556612 | 0.001462 |
| PDK4 | 2.455793 | 0.000655 |
| LPAR1 | 2.404106 | 5.1E-05 |
| UBE2C | -2.29034 | 4.11E-05 |
| CDC25C | -2.00026 | 7.85E-06 |
| EZH2 | -1.92614 | 9.46E-06 |
| CPT1A | 1.853443 | 5.43E-06 |
| HK2 | -1.82701 | 0.004604 |
| BIRC5 | -1.78705 | 0.009927 |
| CASP9 | 1.783598 | 2.79E-05 |
| CXCL12 | 1.674865 | 0.000796 |
| CEACAM8 | -1.64765 | 1.84E-05 |
| MDM2 | -1.60582 | 0.004814 |
| MAD2L1 | -1.59263 | 0.000188 |
| SP1 | -1.57324 | 0.015135 |
| CTNNA1 | -1.52797 | 0.041744 |
| BCL2L1 | -1.39482 | 0.035832 |
| SKP2 | -1.26142 | 0.000351 |
| PDCD4 | -1.22997 | 0.001181 |
| BDNF | -1.14401 | 0.009912 |
| APOBEC3G | 1.11273 | 0.016528 |
| EGF | -1.07919 | 0.003215 |
| S100A4 | 1.062937 | 0.034688 |
| CSPG4 | -1.05192 | 0.029921 |
| PLK1 | -1.04113 | 0.000371 |
| BIN1 | 1.027845 | 0.014641 |
| CALR | -1.01664 | 0.013266 |
| ACP1 | -1.00123 | 0.00066 |
